# Repurposing metformin to manage idiopathic or long COVID Tinnitus: self-report adopting a pathophysiological and pharmacological approach

**DOI:** 10.1007/s10787-023-01421-8

**Published:** 2024-01-31

**Authors:** Mina T. Kelleni

**Affiliations:** Pharmacology Department, College of Medicine, Minia University, Minya, 61111 Egypt

**Keywords:** Tinnitus, Long COVID, Metformin, Caffeine, Adenosine, Adenosine receptors

## Abstract

Chronic tinnitus is a common neurological disorder that affects millions of patients globally with no available successful pharmacotherapy. It can be extremely bothersome to some patients to the extent that it occasionally qualifies as a disability that can hinder them from leading a normal life. In this short communication, the author discusses how he suffered from idiopathic tinnitus and how he managed to adopt a combined pathophysiological and pharmacological approach to the reason for the first time in the medical literature that low-dose metformin might be safely and effectively repurposed to manage at least a subset of tinnitus patients while discussing the potential role of adenosine receptor agonists as potential future tinnitus therapeutics.

Chronic tinnitus (tinnitus as described hereafter) is a common neurological disorder, with ambiguous underlying pathophysiological mechanisms including maladaptive neuroplasticity, that affects at least 10% of all adults and children (Elgandy and Coelho 2022; Gander and Tyler 2022) with a directly proportional increasing percentage in relation to age; up to one-third of those over 65 years, as well as in combat military service members; up to 50% (Kelly Radziwon et al. 2015).

Notably, tinnitus usually accompanies hearing loss which can be caused by numerous factors including drugs (Kelly Radziwon et al. 2015) and it persists for life in 75% of cases. Fortunately, most patients tend to habituate to its persistence, yet at least 10% of patients report a progressive worsening and some become extremely distressed and experience an inability to lead a normal life (Gander and Tyler 2022) and hence it is occasionally rightfully qualifying as a disability.

Interestingly, unilateral tinnitus induced by post-viral labyrinthitis and bilateral tinnitus induced by viral-induced hearing loss were previously described (Figueiredo et al. 2022; Srinivasan 2012). Similarly, SARS CoV-2-induced tinnitus and long COVID tinnitus, were also described with identical ordinary tinnitus characteristics (Figueiredo et al. 2022) and even SARS CoV-2 nucleic acid-based vaccines have also been implicated in the induction of tinnitus (Colizza et al. 2022).

To date, no specific anti-tinnitus drug has been approved, yet many drugs are being tried by clinicians all over the world especially in a quest to assist patients suffering from bothersome or disabling tinnitus, with variable degrees of success (Kim et al. 2021).

I would like to report that I have repurposed metformin, a well-known safe and generic biguanide antihyperglycemic drug that is widely used by clinicians all over the world to mainly manage type 2 diabetes mellitus and overweight in adults as well as in children over 10 years of age (Lentferink et al. 2018; Wang et al. 2017), to address my self-experienced tinnitus that has suddenly and idiopathicly developed bilaterally, though more obvious and intense at the left side, after ignoring to swiftly manage cerumen impaction induced partial left conductive hearing impairment and tinnitus was not reversed after cerumen removal and lasted persistently for almost two months.

Noteworthy, I am in my fifth decade of age, and I do not suffer from any chronic diseases, and fortunately metformin was associated with significant improvement within one week of therapy after I have previously tried a three-week-regimen of using ginkgo biloba extract with negligible improvement, and similarly was the result of zinc supplements, though both were previously described to be successful with some patients (Kim et al. 2021).

Remarkably, I considered to try metformin after experiencing a sudden temporary bothersome increase in tinnitus ringing tone after drinking an unusually ingested cup of coffee at a morning meeting, I have carefully reviewed the complex controversial role of caffeine, an adenosine receptors antagonist, in tinnitus (Aljuaid et al. 2020; Trinidade et al. 2014) that can, like dopamine, possess both excitatory and inhibitory effects (Figueiredo et al. 2021). Interestingly, metformin is known to increase AMP and ADP production in humans and was suggested in an experimental rat model to act as an adenosine receptors activator through an increase in intracellular production of adenosine (Paiva et al. 2009). Importantly, metformin was previously suggested to benefit numerous neurological disorders and multiple potential mechanisms of action were described (Demaré et al. 2021; Li et al. 2022) and its role in tinnitus is currently considered elusive (Bhatt et al. 2022). Moreover, adenosine dopamine neurological interactions are well described (Fuxe et al. 2010; Ross and Venton 2015) and thus it is reasonable to suggest that these interactions might also take place in tinnitus yet to be explored pathophysiology (Dardennes et al. 2013; Lopez-Gonzalez and Esteban-Ortega 2005) aiming at reaching a suitable future therapeutic.

I started with the initial starting dose of metformin 500 mg q.d. in the first week during which I have noticed how tinnitus was rapidly and significantly muffled on the left side for most of the day and almost disappeared on the right side. In the next couple of weeks, I have used it in a dose of 500 mg b.i.d wishing to abolish tinnitus completely and to better manage my mild overweight, yet after a short further improvement, a return of tinnitus, though in a lower tone than the first one, was noticed and obvious and I preferred to return to 500 mg q.d. and the calm muffling was restored to be noted that this low dose has also helped me to adequately control my diet, yet it has also probably encouraged me to crave for more overweight control.

As an explanation and a hypothesis to be further studied, I suggest that increased adenosine levels caused by using metformin might, like its receptors antagonist caffeine, act like a double-edged sword, where increasing the dose might change the response experienced at the affected neurological circuits and pathways. Notably, different concentrations of metformin were previously described to possess and perform different molecular effects (Zhou et al. 2001) and an individualized hormetic dose response regarding metformin purinergic interaction could be also considered (Dattilo et al. 2015).

Tinnitus can be a very frustrating disorder and it affects millions of people all over the world and the numbers of patients have risen sharply amid COVID-19 pandemic and Long COVID Syndrome, and though my self-report has known limitations including the risk of bias and the possibility of individual variations, yet the scientific basis that led me to try metformin as described in this manuscript, i.e., combining pathophysiology and pharmacology, was similar in its structure to the one that has led to Kelleni’s protocol and roadmap (Kelleni 2020, 2022, 2023a, b, c) and likewise I tried it first on myself wishing other patients might benefit from it.

To the best of my knowledge, this is the first academic call to repurpose a low- dose 500 mg metformin q.d. to manage tinnitus and it is reasonable to suggest that at least some tinnitus patients, using metformin 500 mg q.d., might appreciate the calm muffling of its sharp bothersome ringing tone that I have experienced to help them reach their habituation faster, to be reminded that tinnitus might or might not totally disappear afterwards and it is always vigilant to advise the patients not to expect its total disappearance, in my case tinnitus is currently persisting for several months, yet it has been muffled with a faint tone that can be easily ignored (habituated) with fluctuations in this muffling effect especially when I first tried to stop using metformin after reaching it and later when I occasionally forgot to ingest the medication to encounter an unpleasant return of the obvious tinnitus, or an increase in the muffling tone, respectively, and I have decided that it is best for me to continue using metformin, as described, as I truly appreciate the faint tone of the background muffling to which the habituation is almost perfect.

Interestingly, in Egypt and Africa, Long COVID is not common, and tinnitus was never reported by patients in my general clinical practice for over 17 years, probably as tinnitus patients usually consult otolaryngologists, and this has not changed during COVID-19 when I have focused on management of COVID and Post/long COVID patients, yet, and as described before, tinnitus does not differ in its main characteristics regardless of the cause and a trained general practitioner can easily select the eligible patients to try metformin to similarly manage both idiopathic and long COVID tinnitus (Degen et al. 2022).

Taken together, I encourage further studies to assess the potential role of low-dose-metformin, separately or in combination with other safe drugs or nutraceuticals (Elgandy and Coelho 2022; Gomaa et al. 2022; Schloss 2023), to effectively manage at least a subset of tinnitus patients including Long COVID patients. Moreover, research to develop a future adenosine receptor agonist should be considered as a potential drug development area in the quest for a specific anti-tinnitus drug, and a good review of the current adenosine receptor agonists is available (Jacobson et al. 2019).

Finally, I suggest that physicians who manage patients in developing countries where the most modern methods used for its management, though largely futile, are neither available nor affordable, as well as those who manage frustrated patients in developed countries who are suffering the lack of an effective solution, should consider our current call and I suggest that muffling, not complete absence, of tinnitus should be the parameter to inquire about in any tinnitus clinical trial.

## Data Availability

No other scientific data availability for this article.
